# Supplementary material for: Subnanometre-resolution structure of the doublet microtubule reveals new classes of microtubule-associated proteins
Source: Nat Commun. 2017 May 2;8:15035. doi: 10.1038/ncomms15035 (PMC5418579; doi:10.1038/ncomms15035)
Supplement: Supplementary Information — Supplementary Figures, Supplementary Tables, Supplementary Notes and Supplementary References [file ncomms15035-s1.pdf]

Supplementary Materials

Supplementary note 1

The other MIPs

MIP5 densities mainly bind on the outside surface of PF-A12 in our structure. MIP5 actually has a 48-nm repeating unit and there are one slightly larger MIP5a and two MIP5b in 48-nm repeating unit (**Fig. 3g** and **Supplementary Fig. 3b,c**) different from previous study [1]. MIP5 mainly binds between α- and β-tubulin subunits of a dimer (**Supplementary Fig. 3c**). Both MIP5a and 5b are elongated to PF-A11 and bind in between α- and β-tubulin subunits of a dimer in PF-A11 (**Supplementary Fig. 3c**). MIP5a was interacting with MIP3b more intensively than MIP5b (**Fig. 3g**, white arrowheads).

A small density with 48-nm repeating unit bridges PFs-A1 and B10 and connects three tubulin molecules (one tubulin dimer of PF-A1 and α- and β-subunits from two tubulin dimers of PF-B10) (**Supplementary Fig. 3d**).

There are some previously uncharacterized MIP densities in the B-tubule other than MIP3 and MIP7 and named here as minor MIPs (**Fig. 3f**). These minor MIPs are located on the B-tubule fMIPs and thought to be either protein binding on the fMIPs in the B-tubule or part of fMIP proteins.

Supplementary note 2

Filament binding outside the A-tubule

In 48 nm averaged density map, there was a continuous density binding outside between PFs-A2 and A3 and closer to PF-A3 (**Fig. 3g**, blue arrow). This region is known to be the location where the molecular rulers FAP59 and FAP172 are binding [2]. The thickness of this continuous density is similar with a coiled-coil structure (**Supplementary Fig. 4d**). FAP59 and FAP172 homologues in *Tetrahymena* were detected in our doublet sample by mass spectrometry as mentioned above. Taken together, this density is highly likely to be the molecular ruler.

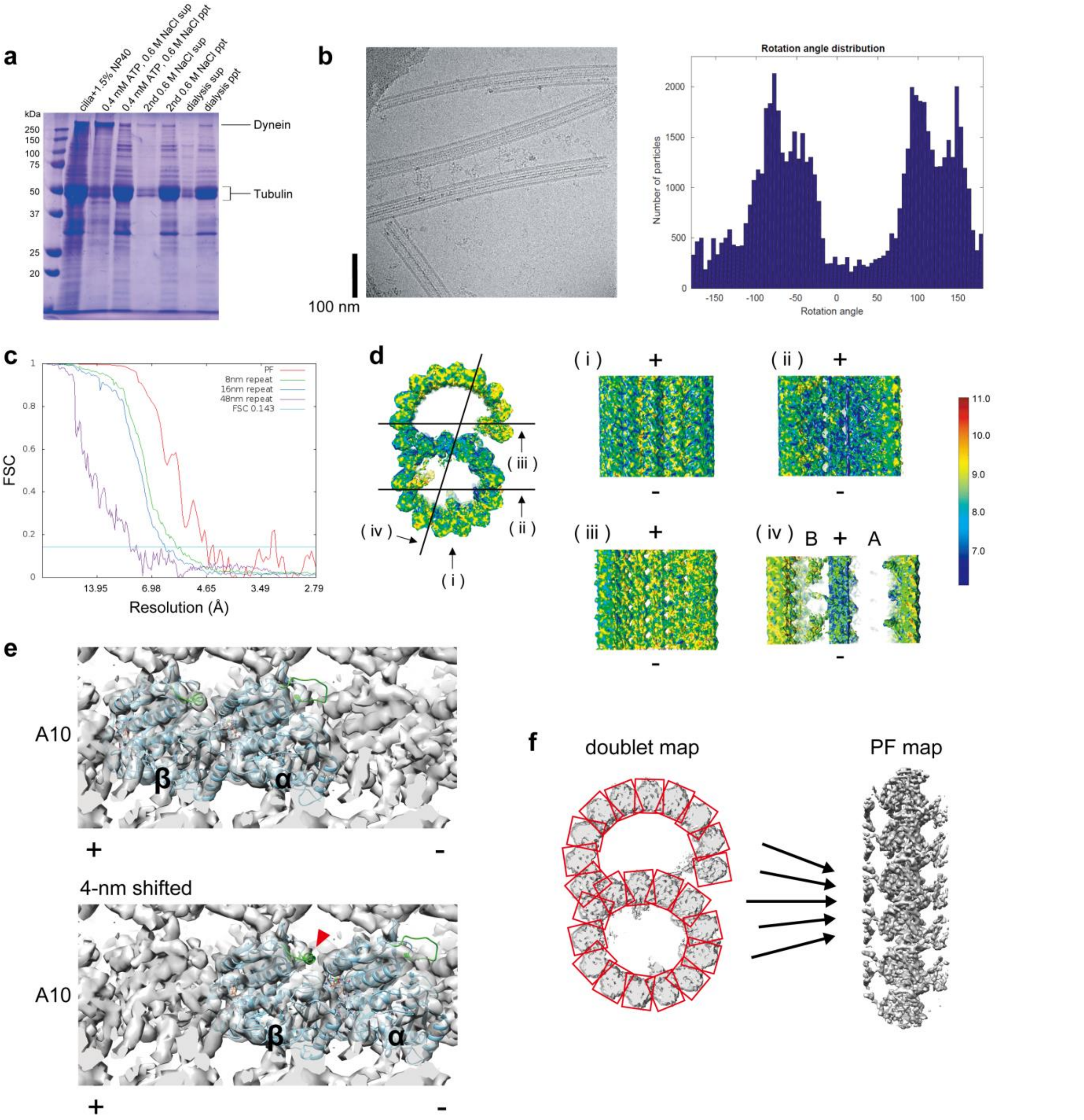

Supplementary Figure 1. Sample preparation and electron microscopy. (a) SDS-PAGE gel of sequentially sub-fractionated doublet. **(b)** Micrographs of doublet fragments in different orientations (left) and azimuthal angular distribution of the particles in the 8-nm density map after alignment (right). **(c)** Gold-standard Fourier shell correlation of the 8, 16, 48-nm, and PF structure. **(d)** Local resolution of the 16-nm averaged density map by ResMap[3] (leftmost panel) and four different views showing outside the A-tubule (i), ribbon region (ii), the B-tubule (iii), and some MIPs (iv). In (iv), A and B means the A- and B-tubules. **(e)** Assignment of α- and β-tubulins in the doublet density map by visual inspection. *Tetrahymena* tubulin model structure was fitted to each protofilament of the 8-nm averaged density map in two different positions which are 4-nm shifted from each other longitudinally. PF-A10, whose assignment was not determined by previous research[1] is shown here as an example. Tubulin regions (aa 36-50), which form a loop in α-tubulin and an α-helix in β-tubulin, are colored green. In the 4-nm shifted model (lower row), the α-helix is sticking out from the density map (indicated by red arrowhead). Cross-correlation coefficient values between tubulin and density map before and after 4-nm shift were 0.9217 and 0.9075, respectively. **(f)** Averaging of PF from doublet density map. Each subvolume of PF (red boxes) centering in the middle of a tubulin dimer was further boxed out from the doublet map (left) and averaged to get the PF map (right).

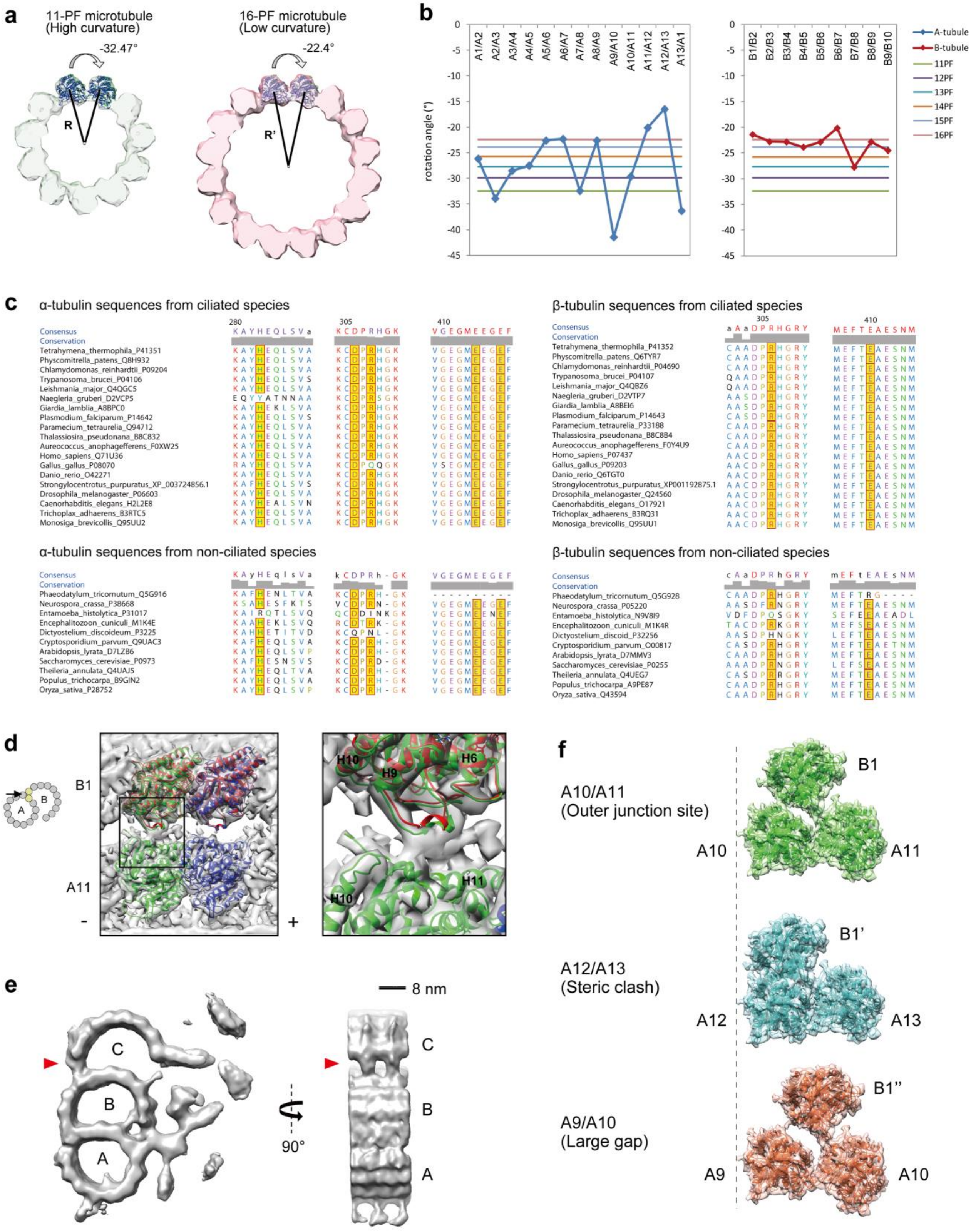

Supplementary Figure 2. Data related to doublet tubulin lattice. (a) Relationship between curvature of microtubule lattice and rotation angle of neighboring tubulin molecules. For high curvature microtubule (small number of PFs), absolute value of rotation angle of tubulin is larger with smaller radius (R). For low curvature microtubule (large number of PFs), absolute value of rotation angle of tubulin is smaller with larger radius (R'). Density maps shown are EMD-5191 and 5196 [4] and gaussian

filtered for visualization. Rotation angle values are also adopted from previous study [4]. **(b)** Plots of rotation angles between adjacent PF models (see also **Supplementary Table 1**) with lines showing angle values from 11- to 16-PFs singlet microtubules[4]. **(c)** The multiple sequence alignment of tubulin residues involved in non-canonical tubulin-tubulin interactions at the outer junction (highlighted in yellow). *Tetrahymena* amino acid numbers are indicated. **(d)** Tubulin model structures fitted to PF-B1 density. The view is the same with **Fig. 2e**. Green and blue, model after Phenix refinement[5]; red, model before refinement. Cross-correlation values between tubulin model structures and the density map are 0.9316 and 0.9412 before and after refinement. **(e)** Structure of triplet from previous cryo-electron tomography study (EMD-5252 [6]). The part of the C-tubule attachment at the outer junction side (indicated by red arrowheads) suggests the existence of non-tubulin proteins. **(f)** Hypothetical outer junction structures formed on the other PF pairs of the A-tubule. The outer junction tubulin model structure (top row) was divided into two regions (A10-tubulin and A11/B1-tubulins) and fitted to other neighboring PFs in the doublet structure. For PF pair-A12/A13 with a low curvature, steric clash occurred between A12- and B1'-tubulins (middle row). For PF pair-A9/A10 with a high curvature, no salt bridge was formed between A9- and B1"-tubulins as determined by PDBePISA [7].

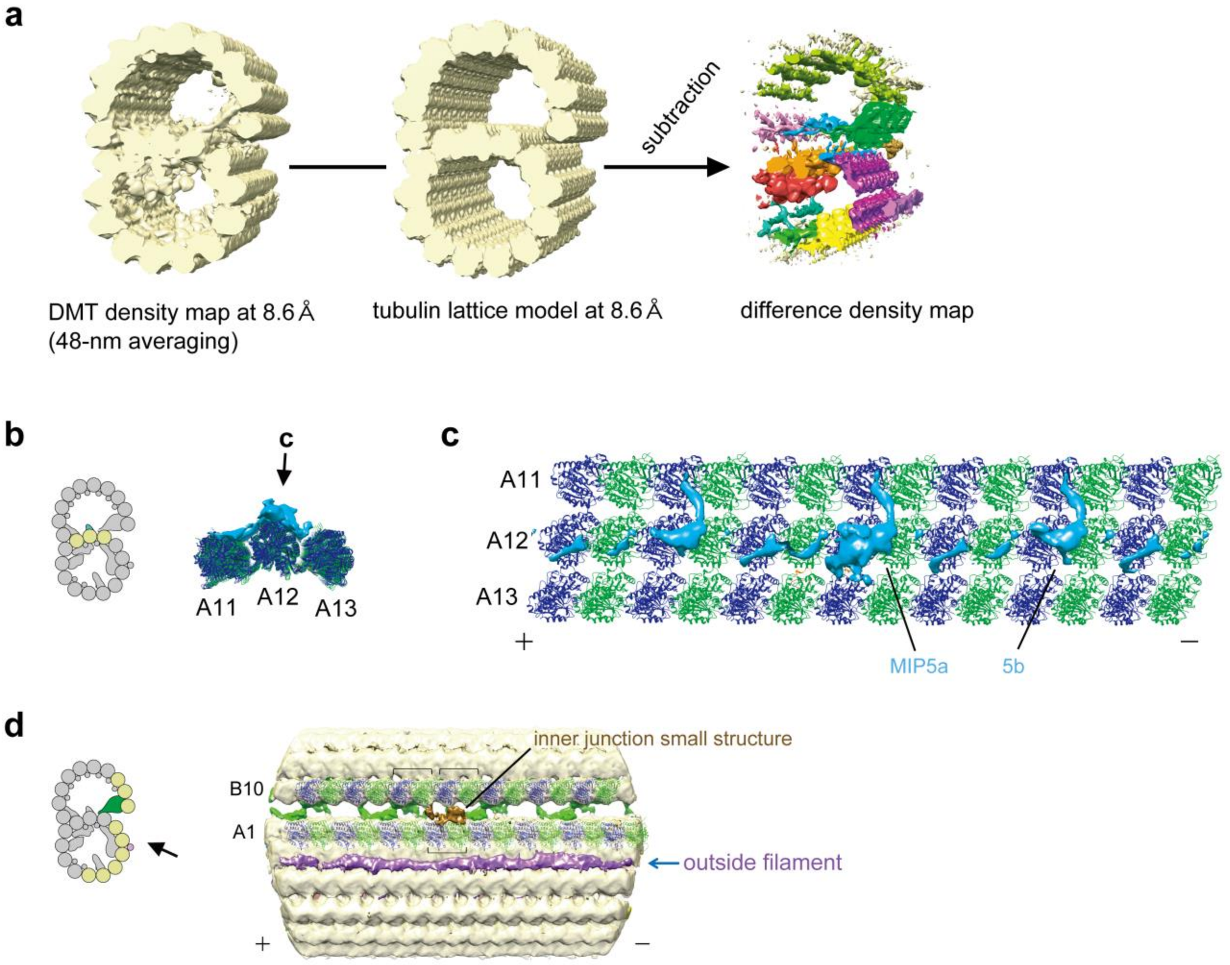

Supplementary Figure 3. Related to MIPs densities. (a) Procedure of the subtraction. The tubulin lattice model at 8.6 Å was generated using molmap function of UCSF Chimera [8] and subtracted from 48-nm averaged density map to create the difference map. Difference map was colored manually based on the location, and segmented. The density map after the segmentation is shown in **Fig. 7a**. Similar procedure was performed to generate the difference map from the 16-nm averaged structure. **(b,c)** Views of interactions between MIP5 and tubulin lattice in 48-nm averaging. **(d)** View showing interactions between tubulin molecules and small inner junction structure in 48-nm averaged structure. Outside filament is indicated by blue arrow. Views in (b,d) are from the tip of cilia (+ end). (+) and (-) indicate the polarities of PFs in (c,d) and brackets in (d) indicate the tubulin dimers.

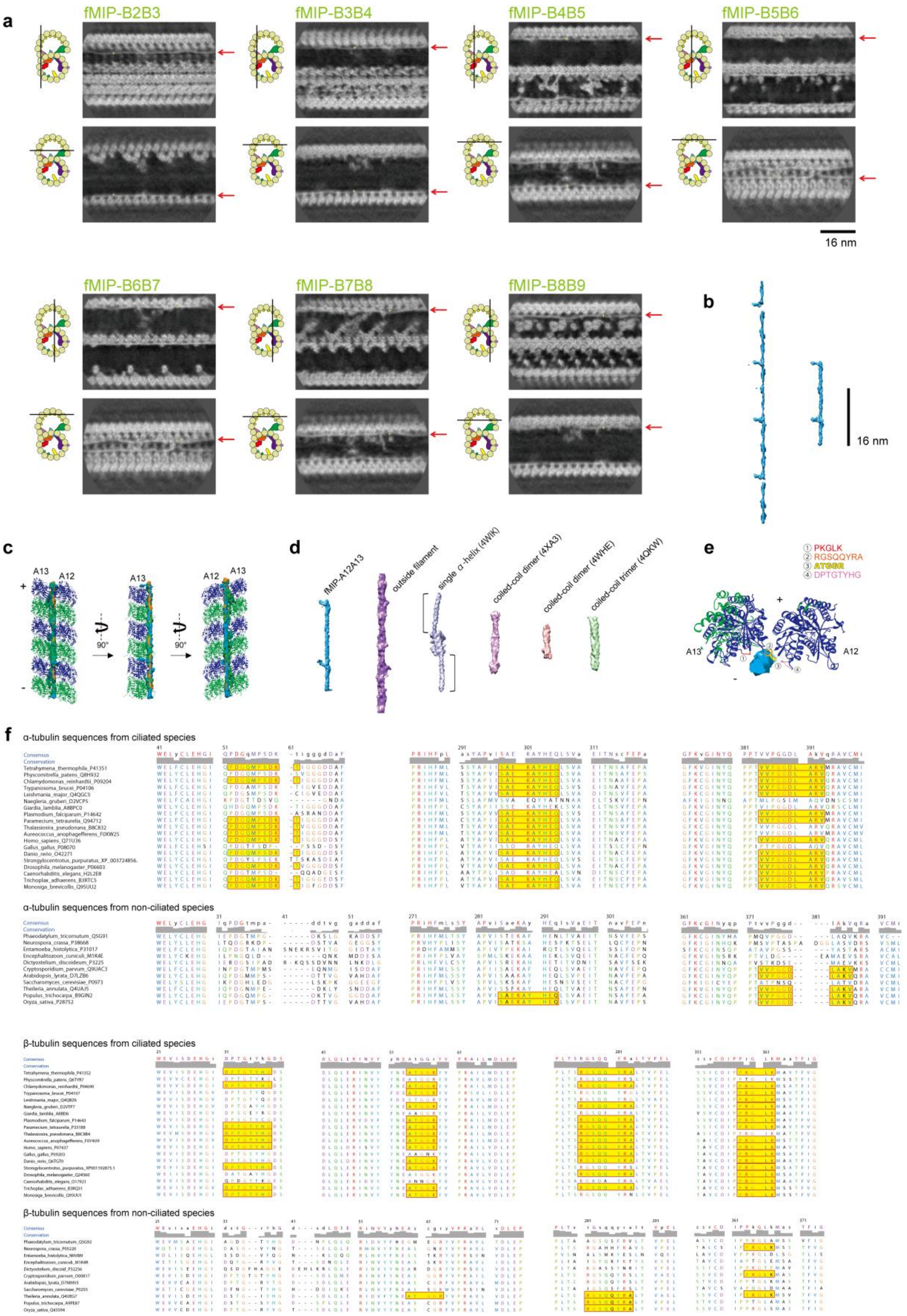

Supplemental Figure 4. Data related to fMIPs. (a) Longitudinal sections of fMIPs in the B-tubule (indicated by red arrows). Black lines in cartoons on the left show the sections. **(b)** Comparison of

fMIP-A12A13 structures in 48-nm map (left) and 16-nm map (right). Protrusions of fMIP-A12A13 appear similar in both averaging map, showing that fMIP-A12A13 has 16-nm periodicity. **(c)** Comparison of fMIPs location relative to tubulin lattice. fMIPs in the A-tubule are superposed on the PFs-A12/A13 tubulin lattice. Note that 16-nm averaged structures were used here. **(d)** Comparison of fMIP-A12A13 in 16-nm structure and outside filament in 48-nm structure from our density map and 8 Å model density maps of single α-helix (PDB: 4WIK), coiled coil dimer (PDB: 4XA3, 4WHE) and coiled-coil trimer (PDB: 4QKW). Single α-helix regions of PDB: 4WIK are indicated by brackets. **(e)** Four regions of β-tubulins from PFs-A12 and A13 possibly interacting with fMIP-A12A13. View is the same with **Fig. 5c**. **(f)** Sequence alignment results of α- and β-tubulins from both ciliated species and non-ciliated species. The regions involved in binding to fMIPs (PDGQMPSDKT, SAEKAYHEQ, VVPGGDLAKV, DPTGTYHG, ATGG(R/K), RGSQQYRA and P(K/R)GLK) are highlighted in yellow.

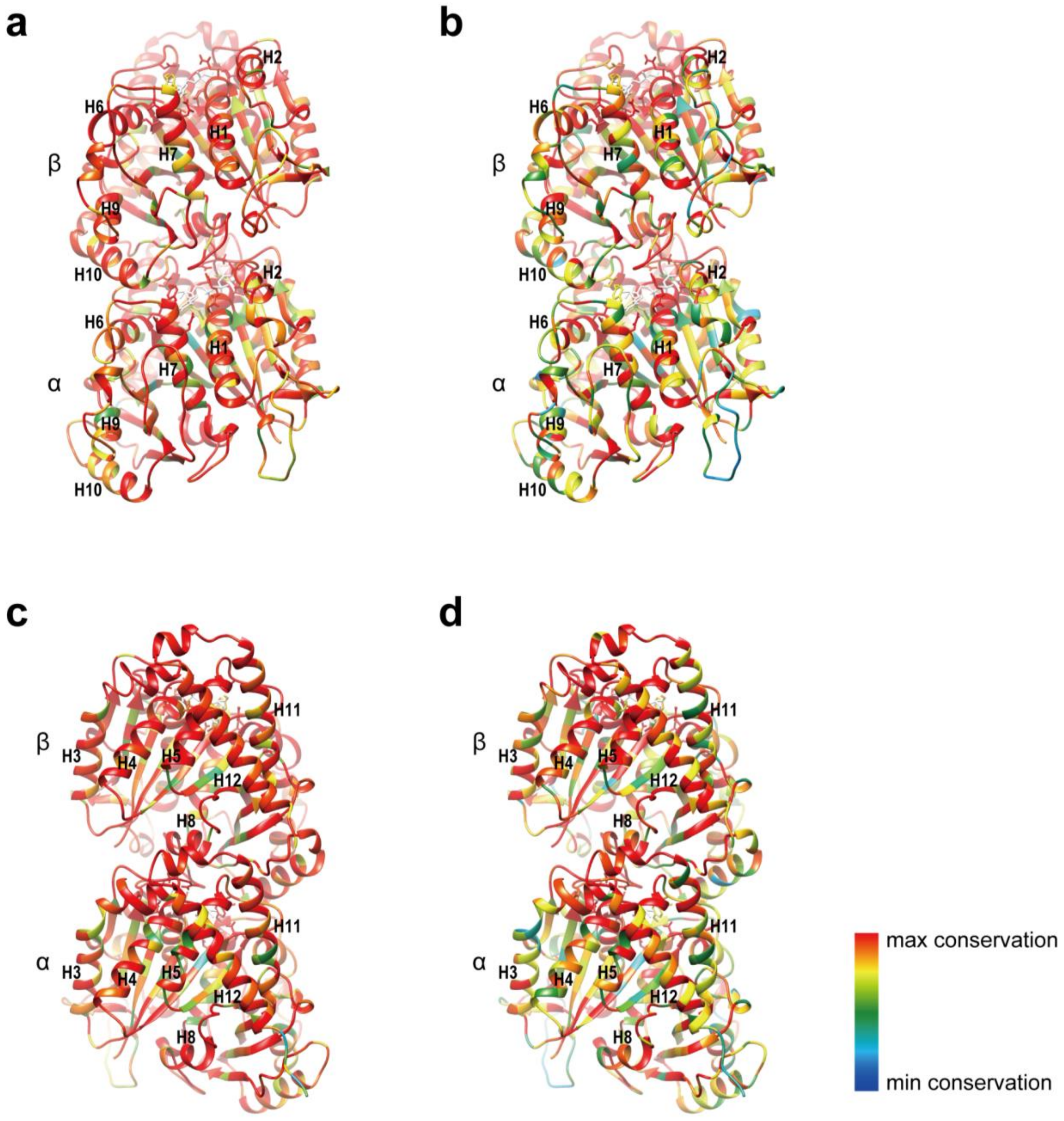

Supplemental Figure 5. Conservation of the sequence of the tubulin. Sequence conservation of tubulin molecules viewed from inside (a,b) and outside (c,d). Here, we performed multiple sequence alignments of α- and β-tubulins from 35 ciliated organisms (a,c) and 15 non-ciliated organisms (b,d) by Clustal Omega [9]. After that, we calculated the identity histogram of each position in the multiple sequence alignment, which defines what proportion of the sequences have the most prevalent non gap character per alignment column by UCSF Chimera [8]. The identity histogram of each residue was visualized on our modeled tubulin dimer structure by UCSF Chimera [8]. Tubulin surfaces of ciliated organisms (a,c) are more conserved compared with that of non-ciliated ones (b,d). α- and β-tubulins and the α-helix numbers are indicated. Species names and protein IDs of tubulin used for conservation analysis are listed in **Supplementary Table 5.**

SUPPLEMENTARY TABLES

Supplementary Table 1. Curvature between protofilaments

	Rotation (°)	Z-shift (Å)	Theoretical microtubule with PF number of
**A1/A2 (MIP6)**	-26.14	9.65	13.8
**A2/A3 (MIP6)**	-33.89	9.68	10.6
**A3/A4 (MIP6)**	-28.49	9.48	12.6
**A4/A5 (MIP1)**	-27.49	8.78	13.1
**A5/A6 (MIP1)**	-22.6	9.44	15.9
**A6/A7 (fMIP)**	-22.29	9.35	16.2
**A7/A8 (fMIP)**	-32.41	9.58	11.1
**A8/A9 (MIP2)**	-22.6	9.44	15.9
**A9/A10 (MIP2, seam)**	-41.43	31.64	8.69
**A10/A11 (MIP2, MIP4, MIP7)**	-29.62	9.33	12.2
**A11/A12 (fMIP)**	-20.09	9.31	17.9
**A12/A13 (fMIP)**	-16.49	9.40	21.8
**A13/A1 (MIP6)**	-36.3	9.49	9.92
**B1/B2 (MIP7)**	-21.39	9.58	16.8
**B2/B3 (fMIP)**	-22.79	9.24	15.8
**B3/B4 (fMIP)**	-22.84	9.19	15.8
**B4/B5 (fMIP)**	-23.86	9.08	15.1
**B5/B6 (fMIP)**	-22.87	9.06	15.7
**B6/B7 (fMIP)**	-20.15	9.03	17.9
**B7/B8 (fMIP)**	-27.76	9.40	13.0
**B8/B9 (fMIP)**	-22.81	8.98	15.8
**B9/B10 (MIP3)**	-24.5	9.17	14.7

Supplementary Table 2. Interface calculation between tubulins in the outer junctions by Haddock

	Longitudinal interaction		Lateral interaction					
	Intra dimer	Inter dimer	A10_α A11_α	A10_β A11_β	A10_α B1_α	A10_β B1_β	A11_α B1_α	A11_β B1_β
**HADDOCK score**	-292.3	-209.6	-103.2	-87.8	-66.3	-55.2	-48.3	-40
**Van der Waals energy ($kcal.mol^{-1}$)**	-136.9	-146.4	-34.6	-31.6	-15.8	-13	-12.6	-5.2
**Electrostatic energy ($kcal.mol^{-1}$)**	-636.6	-349.4	-264.9	-225.1	-185	-140.4	-1.6	-0.5
**Desolvation energy ($kcal.mol^{-1}$)**	-28	6.6	-15.6	-11.2	-13.5	-14.1	-35.4	-34.7
**Buried Surface Area ($Å^2$)**	3880.9	3739.9	1205.9	1133	688.6	462.1	351.7	137.5

Supplementary Table 3. globular MIPs' repeating unit, binding pattern, and interactions with other MIPs

	Subtypes	Repeating units	Binding locations	Interactions with other MIPs
**MIP1**	MIP1a	16 nm	A4/A5●†‡, **A5†‡,** A5/A6●†‡	MIP1b, MIP6a, fMIP-A6A7
	MIP1b	16 nm	A4/A5●†‡, **A5†‡,** A5/A6●†‡	MIP1a, MIP6b
**MIP2**	MIP2a	48 nm	**A9**†‡, A9/A10●§†‡, A11(β)	MIP4a, MIP7 fMIP-A7A8
	MIP2b	48 nm	A8/A9●†, **A9**†‡, A9/A10●§†‡	MIP4c
	MIP2c	48 nm	A8/A9†, **A9†**, A9/A10●§†‡	MIP4d fMIP-A7A8
**MIP3**	MIP3a	16 nm	A12/A13*●†, A13*(α), A13/A1*●‡, B9†‡, B10†‡	MIP3b, fMIP-B8B9
	MIP3b	16 nm	A12/A13*●†, A13*(α), A13/A1*●‡, B9/B10●‡	MIP3a, MIP5a, 5b
**MIP4**	MIP4a	48 nm	A10/A11●†, **A11†, A12**†	MIP2a fMIP-A11A12 fMIP-A12A13
	MIP4b	48 nm	A10/A11●‡, **A11‡, A12†‡**	fMIP-A11A12
	MIP4c	48 nm	A10/A11●†‡, **A11†‡, A12†‡**	MIP2b, MIP4d, MIP6a, MIP7, fMIP-A11A12 fMIP-A12A13
	MIP4d	48 nm	A10/A11●†‡, **A11†‡, A12†**	MIP2c, MIP4c fMIP-A11A12 fMIP-A12A13
**MIP5**	MIP5a	48 nm	A11*†, **A12*†**	MIP3a, 3b
	MIP5b	48 nm	A11*†, **A12*†**	MIP3b
**MIP6**	MIP6a	16 nm	A1†‡, A2†‡, A3†‡, A13†	MIP1a, MIP4c, MIP4d fMIP-A11A12 fMIP-A12A13
	MIP6b	16 nm	A1†‡, A2†‡, A3†‡, A13†	MIP1b
**MIP7**		16 nm	A11*†‡, B1†‡, B1/B2†‡	MIP2a, MIP4c
**minor MIPs**		48 nm		fMIP-B5B6, fMIP-B6B7, fMIP-B7B8, fMIP-B8B9
**Inner junction small structure**		48 nm	A1*†, B10(α, β)	no

main protofilament in **bold**; *, binding to outside surface; †, binding between subunits of one tubulin dimer; ‡, binding between two tubulin dimers; ●, binding between protofilaments; §, binding at the seam

Supplementary Table 4. fMIPs properties: morphology, interaction and possible repeating units

Filaments	Curvature	Continuity	Interactions with other MIPs	Insertions to tubulin lattice	Possible repeating units
fMIP-A6A7	mostly straight, sudden curve at one end	one gap in 48 nm	one of MIP1a in 48 nm unit, fMIP-A7A8	yes	48 nm
fMIP-A7A8	curved	one gap in 48 nm	MIP2a, 2c, fMIP-A6A7	yes	48 nm
fMIP-A11A12	straight	two gaps in 48nm	MIP4, MIP6a	yes	48 nm
fMIP-A12A13	very straight	continuous	MIP6a (every 16nm), MIP4a, 4c, 4d	yes (every 16 nm)	16nm
fMIP-B2B3	curved	continuous	no	no obvious insertion	48 nm
fMIP-B3B4	curved	continuous	no	no obvious insertion	48 nm
fMIP-B4B5	curved	continuous	no	no obvious insertion	48nm
fMIP-B5B6	curved	one gap in 48 nm	minor MIPs fMIP-B6B7	no obvious insertion	48nm
fMIP-B6B7	curved	continuous	minor MIP fMIP-B5B6, fMIP-B7B8	yes	48nm
fMIP-B7B8	curved	continuous	minor MIP fMIP-B6B7, fMIP-B8B9	yes	48nm
fMIP-B8B9	curved	continuous	two of MIP3a in 48 nm unit, fMIP-B7B8	yes	48nm

Supplementary Table 5. Species and protein IDs used for conservation analysis

Organism	α-tubulin ID	β-tubulin ID	Motile cilia	Non-motile cilia
Tetrahymena thermophilia	P41351	P41352	+	-
Trypanosome brucei	P04106	P04107	+	-
Micromonas pusilla (Picoplanktonic green alga)	C1MS44	C1ML15	+	-
Chlamydomonas reinhardtii	P09204	O04386	+	-
Gallus gallus	P02552	P09203	+	+
Giardia lamblia	Q9U014	P05304	+	-
Lottia gigantea (Giant owl limpet)	V4B157	V4ATA4	+	+
Drosophila melanogaster (Fruit fly)	P06603	Q24560	+	+
Homo sapiens	Q71U36	P07437	+	+
Danio renio	Q6DC49	Q6NW90	+	+
Amphimedon queenslandica	I1FP07	I1GBV3	+	-
Anopheles gambiae	Q7PUE2	Q7PSI4	+	+
Apis mellifera (Honeybee)	A0A088ANF0	A0A087ZSC1	+	+
Branchiostoma floridae	C3Z395	C3Y2F5	+	+
Brugia malayi	A0A0K0JTQ1	A0A0K0JFY1	-	+
Caenorhabditis elegans	O18688	P12456	-	+
Capitella teleta (Polychaete worm)	R7T6Z8	R7TKU3	+	+
Daphnia pulex (Water flea)	E9GAV8	E9GER1	-	+
Nematostella vectensis (Starlet sea anemone)	A7RUT1	A7S8I3	+	+
Schistosoma mansoni (Blood fluke)	Q26595	C4QIC0	+	+
Strongylocentrotus purpuratus	W4ZJ54	W4X9L8	+	+
Tribolium castaneum (Red flour beetle)	D6WBF0	D6WPR3	-	+
Trichoplax adhaerens	B3RTC1	B3RQ31	+	?
Monosiga brevicollis (Choanoflagellate)	Q95UU2	A9UTT4	+	-
Batrachochytrium dendrobatidis (Frog chytrid fungus)	F4NRM5	F4P7H8	+	-
Physcomitrella patens subsp. patens (Moss)	Q8H932	Q6TYR5	+	-
Selaginella moellendorffii (Spikemoss)	D8QVP0	D8TAZ0	+	-
Emiliania huxleyi (Pontosphaera huxleyi)	R1E539	R1BPR0	+	-
Trichomonas vaginalis	A2E0M4	Q27116	+	-
Naegleria gruberi (Amoeba)	P11237	D2VTP7	+	-
Bigelowiella natans	Q5YEV0	Q9LD24	+	-
Plasmodium falciparum	P14642	P14643	+	-
Guillardia theta (Cryptomonas phi)	Q9SCC8	Q9SEV2	+	-
Toxoplasma gondii	P10873	P10878	+	-
Paramecium tetraurelia	Q94712	P33188	+	-
Aspergillus niger	A2Q8U9	A2QQP0	-	-

Laccaria bicolor	B0CXU8	B0DHG8	-	-
Saccharomyces cerevisiae	P09733	P02557	-	-
Ustilago maydis	Q9HDT1	Q4P235	-	-
Dictyostelium discoideum	P32255	P32256	-	-
Entamoeba histolytica	P31017	C4LUJ0	-	-
Arabidopsis thaliana	P11139	P12411	-	-
Cyanidioschyzon merolae	Q84KQ3	Q84KQ2	-	-
Populus trichocarpa	A9P817	A9PEU3	-	-
Phaeodactylum tricornutum	B7G0C3	B5Y3W7	-	-
Encephalitozoon cuniculi	Q8SRI6	Q8SS99	-	-
Oryza sativa	P28752	Q43594	-	-
Theileria annulata	Q4UGQ9	Q4UEG7	-	-
Neurospora crassa	P38668	P05220	-	-
Cryptosporidium parvum	Q9UAC3	Q8MPB3	-	-

Supplementary References

1. Maheshwari, A. et al. alpha- and beta-Tubulin Lattice of the Axonemal Microtubule Doublet and Binding Proteins Revealed by Single Particle Cryo-Electron Microscopy and Tomography. *Structure* **23**, 1584-95 (2015).
2. Oda, T., Yanagisawa, H., Kamiya, R. & Kikkawa, M. A molecular ruler determines the repeat length in eukaryotic cilia and flagella. *Science* **346**, 857-60 (2014).
3. Kucukelbir, A., Sigworth, F.J. & Tagare, H.D. Quantifying the local resolution of cryo-EM density maps. *Nat Methods* **11**, 63-5 (2014).
4. Sui, H. & Downing, K.H. Structural basis of interprotofilament interaction and lateral deformation of microtubules. *Structure* **18**, 1022-31 (2010).
5. Adams, P.D. et al. PHENIX: a comprehensive Python-based system for macromolecular structure solution. *Acta Crystallogr D Biol Crystallogr* **66**, 213-21 (2010).
6. Li, S., Fernandez, J.J., Marshall, W.F. & Agard, D.A. Three-dimensional structure of basal body triplet revealed by electron cryo-tomography. *EMBO J* **31**, 552-62 (2012).
7. Krissinel, E. & Henrick, K. Inference of macromolecular assemblies from crystalline state. *J Mol Biol* **372**, 774-97 (2007).
8. Pettersen, E.F. et al. UCSF Chimera--a visualization system for exploratory research and analysis. *J Comput Chem* **25**, 1605-12 (2004).
9. Sievers, F. et al. Fast, scalable generation of high-quality protein multiple sequence alignments using Clustal Omega. *Mol Syst Biol* **7**, 539 (2011).
